# Supplementary material for: Microbe transmission from pet shop to lab-reared zebrafish reveals a pathogenic birnavirus
Source: PLoS Biol. 2024 May 30;22(5):e3002606. doi: 10.1371/journal.pbio.3002606 (PMC11139271; doi:10.1371/journal.pbio.3002606)
Supplement: S1 Fig — Differential gene expression was measured for the 3 replicates from each pet trade source compared to the 3 laboratory replicates (data in S4 File). Log2 fold change values are plotted for a selected set of antiviral genes with adjusted p-values <0.1. (PDF) [file pbio.3002606.s001.pdf]

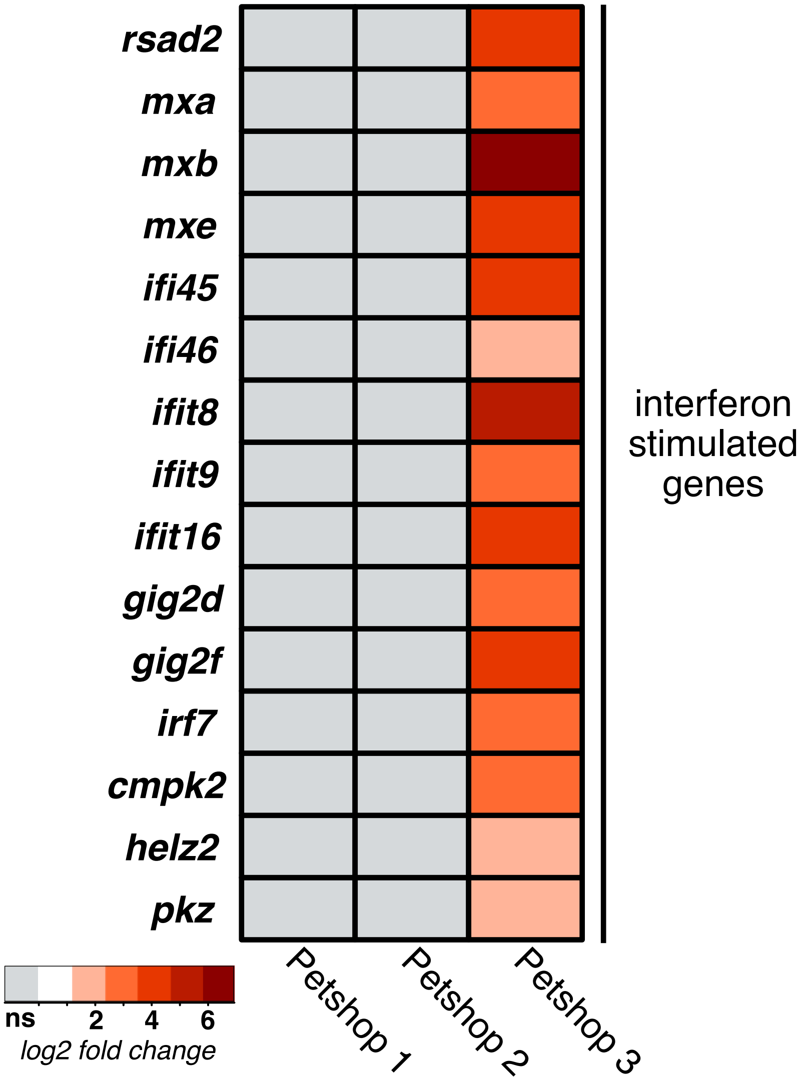

S1 Figure. Antiviral gene expression in zebrafish from pet trade sources compared to control zebrafish from the laboratory. Differential gene expression was measured for the three replicates from each pet trade source compared to the three laboratory replicates (data in S4 File). Log2 fold change values are plotted for a selected set of antiviral genes with adjusted p-values < 0.1.
