# Supplementary material for: Microbe transmission from pet shop to lab-reared zebrafish reveals a pathogenic birnavirus
Source: PLoS Biol. 2024 May 30;22(5):e3002606. doi: 10.1371/journal.pbio.3002606 (PMC11139271; doi:10.1371/journal.pbio.3002606)
Supplement: S2 Fig — (A) Diagram of cohousing experiment. Tanks were seeded with adult zebrafish from the laboratory and pet trade source 3, as in Fig 2. (B) Transmission of microbes from pet trade to cohoused laboratory zebrafish inferred from host and microbe transcript abundances. Intestines from 2 donor pet trade zebrafish and 2 laboratory recipient fish were sampled after 1 month of cohousing. Control zebrafish are the same as shown in Fig 2. Columns show estimated transcript abundances in intestine samples from individual zebrafish. (PDF) [file pbio.3002606.s002.pdf]

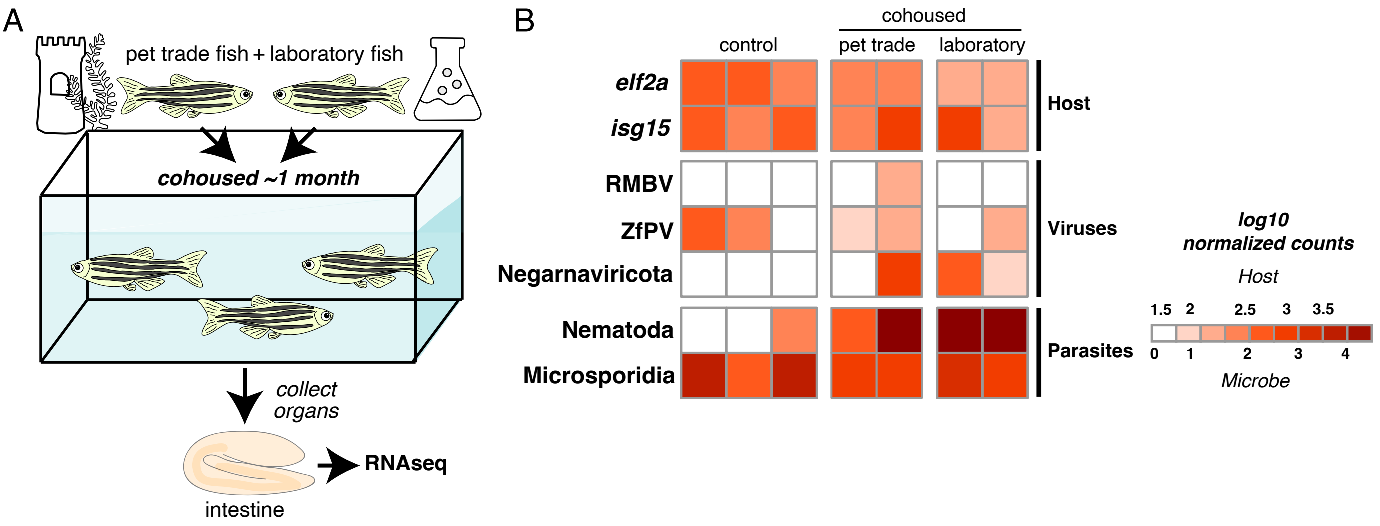

S2 Figure. Zebrafish from the pet trade can harbor RMBV without transmitting to cohoused zebrafish from the laboratory. (A) Diagram of cohousing experiment. Tanks were seeded with adult zebrafish from the laboratory and pet trade source 3, as in Figure 2. (B) Transmission of microbes from pet trade to cohoused laboratory zebrafish inferred from host and microbe transcript abundances. Intestines from two donor pet trade zebrafish and two laboratory recipient fish were sampled after one month of cohousing. Control zebrafish are the same as shown in Figure 2. Columns show estimated transcript abundances in intestine samples from individual zebrafish.
