# Supplementary material for: Microbe transmission from pet shop to lab-reared zebrafish reveals a pathogenic birnavirus
Source: PLoS Biol. 2024 May 30;22(5):e3002606. doi: 10.1371/journal.pbio.3002606 (PMC11139271; doi:10.1371/journal.pbio.3002606)
Supplement: S5 File — (PDF) [file pbio.3002606.s007.pdf]

A

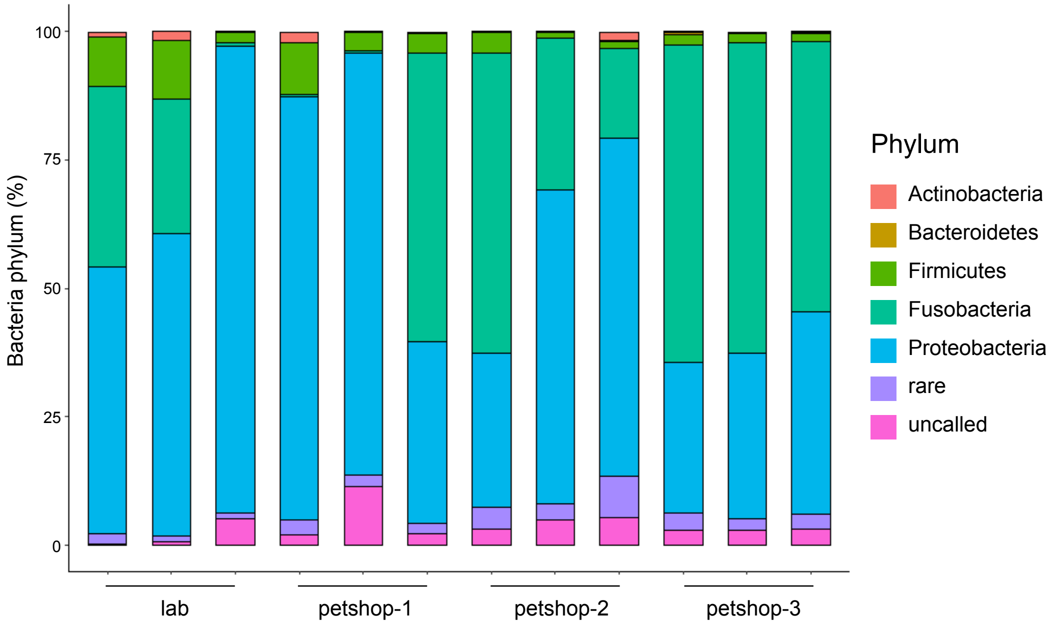

B

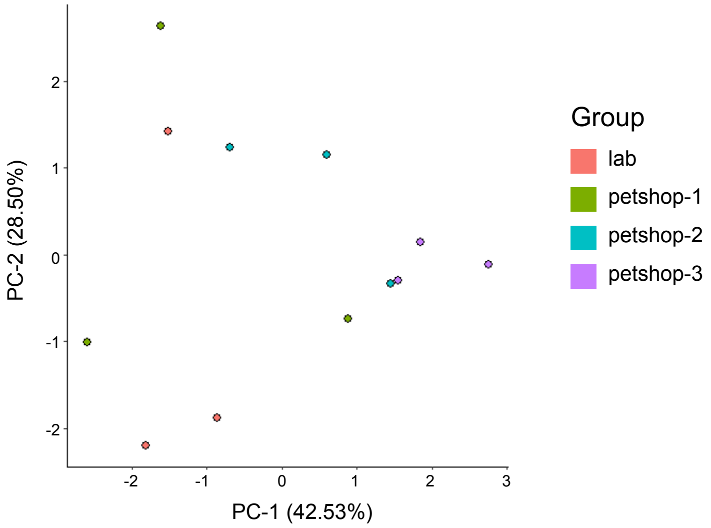

Supplemental file 5 Gut microbiome composition of lab and pet store zebrafish. A) bar graphs illustrating bacteria relative abundance by phylum. Relative abundance was calculated form normalized read counts from each sample. Each column represents an individual fish intestine sample. N = 3 for each group. Note large degree of variation between individuals and between groups. B) Principal component analysis using relative abundances of each bacteria phylum per individual zebrafish. PC-1 and PC-2 combined account for ~ 70% of variation between samples. Note large variation within and between groups. Small sample size per group (n = 3) prevented us from plotting a 95% confidence ellipse for each group. A & B) generated with ggplot2 in R studio.
