# Supplementary material for: Microbe transmission from pet shop to lab-reared zebrafish reveals a pathogenic birnavirus
Source: PLoS Biol. 2024 May 30;22(5):e3002606. doi: 10.1371/journal.pbio.3002606 (PMC11139271; doi:10.1371/journal.pbio.3002606)
Supplement: Attachment — Submitted filename: Response_to_Reviwers.pdf [file pbio.3002606.s010.pdf]

We thank the reviewers for the favorable reception of our Discovery Report and address the constructive criticism raised in each point below. The suggestions are helping to guide follow-up work that we anticipate submitting as an Update Article in support of this manuscript.

Reviewer #1: This is a unique contribution from a team with a track record of discovery innovating zebrafish-virus infection models. In this case authors use metagenomic sequencing and co-housing experiments to discover novel zebrafish-associated microbes and virus infections. The authors report Rocky Mountain birnavirus (RBMV) as a natural zebrafish pathogen, and that lab animals develop disease symptoms when co-housed with infected animals from a pet shop. In the same way that zebrafish infection using the natural fish pathogen Mycobacterium marinum has evolved to become a paradigm for studying human tuberculosis and natural host-pathogen interactions, the research avenue presented here looking for natural virus infections of zebrafish can similarly inspire.

Many of my comments are outside the scope of this first discovery report, but interesting to consider for this or future work.

We agree, and will use this feedback to guide ongoing and future work on RMBV.

To identify RBMV, why was sequencing of intestinal RNA first performed? Can infection biology be used to support inferences from omics or co-housing experiments (where evidence can be indirect). Is isolating RBMV from infected zebrafish not possible at this stage? If possible, it would be valuable to test Koch's postulates eg. isolate virus from infected zebrafish and infect lab animal, in this way directly asking if RBMV is causing disease.

Given the prevalence of enteric viruses we previously characterized in zebrafish along with the general importance of the gut as a host-microbe interface, we reasoned that surveying intestinal tissues was a likely place to discover potentially infectious microbes. After discovering the virus in the intestine, we broadened our measurements to include the kidney and spleen as potential sites of dissemination.

We agree that testing Koch’s postulates is a priority for ongoing future work. Our surveys in this effort were by necessity destructive to the virus-containing tissues, and our preliminary efforts were unsuccessful in isolating infectious virus particles from the remaining frozen carcasses months later.

Our current efforts include a deeper survey for live infected fish from additional pet stores (see response to the next comment) and some encouraging progress towards launching an infectious clone of RMBV in zebrafish tissue culture cells. However, this work will take substantial time to ensure that the assays are robust and reproducible.

We appreciate the reviewer’s recognition that co-housing and related sequence analysis offers a valuable strategy for virus discovery from otherwise complex metagenomic data.

Why are pet shop zebrafish resistant to hemorrhaging but lab zebrafish are not? What if infected zebrafish are co-housed with other uninfected pet shop zebrafish (eg. from sources where RBMV was not identified)? I understand that experiments involving adult fish are not trivial, but would appreciate some perspectives on this.

Great question! When the pet store fish were purchased they appeared healthy with no outward signs of disease. We consider several non-exclusive possibilities that could explain why pet shop zebrafish appear resistant to the severe symptoms exhibited by lab fish, including genetic background, prior exposure to birnavirus, or prior or current exposure to other environmental stimuli of the immune system (microbiome, etc) that modify a birnavirus infection. For example, we cannot exclude the possibility that pet store fish at one time exhibited lesions, recovered, and then served as reservoirs to infect laboratory fish. As the reviewer suggests, we need to conduct additional experiments to distinguish between these possibilities, including identifying uninfected pet store zebrafish, generating “lab” versions of pet store genotypes, and surveying transmission between lab and pet store strains. We hope to conduct these experiments in the future.

Because we do not currently have IACUC approval to do these experiments, we instead include some additional co-housing experiments which extend our initial findings. In the revised manuscript we now provide the results from a second co-housing experiment (Figure S2). In this experiment, intestines from two pet trade (from the same source as in the first co-housing experiment) and two laboratory fish were sampled by RNAseq after a month of co-housing as described in the first experiment. In the newly presented experiment, one of the two pet trade fish was positive for RMBV. Although we only sampled 2 of the 10 laboratory fish from this co-housing experiment, neither laboratory fish was positive for RMBV at the time of sampling and none of the fish in the tank exhibited signs of hemorrhaging. These results support the notion that fish can be carriers of RMBV without transmitting infection. We also share below the preliminary results from a third and ongoing co-housing experiment, in which pet trade fish from a different pet store source (also known to be a source of RMBV) were co-housed with laboratory fish. We have not seen hemorrhaging in laboratory fish in this experiment, but 1/4 fish sampled so far had high levels of RMBV RNA suggesting transmission from the pet trade fish (Reviewer Figure 1). In summary, we believe that a spectrum of transmission, antiviral responses, and hemorrhagic symptoms are possible in a natural RMBV infection scenario. We discuss more possibilities below.

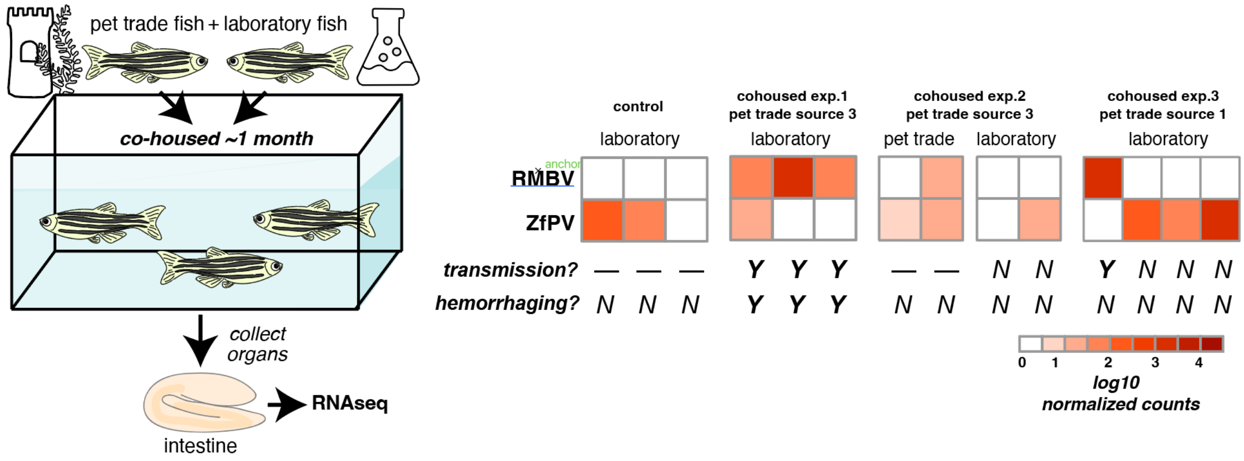

Reviewer Figure 1. A summary of all pet trade and laboratory co-housing experiments. Fish from three co-housing experiments were sampled by RNAseq for RMBV. In experiment 1, we observed transmission from pet trade to laboratory fish that had hemorrhaging symptoms. In experiment 2, we observed no transmission. In experiment 3, we observed transmission of RMBV, but no hemorrhaging symptoms. We present data from experiments 1 and 2 in the revised manuscript, but our ongoing work related to the third experiment is not yet finalized for publication.

3/15 lab zebrafish show signs of infection from the co-housing experiments? If 12/15 lab zebrafish do not become infected, should the authors consider to modify their conclusions?

We have revised our methods section to clarify that the co-housing tanks contained 10 laboratory zebrafish and 5 pet trade zebrafish. Thus, in the first co-housing experiment we detected RMBV in 3/10 laboratory zebrafish that also exhibited hemorrhaging. We did not sample the 7 asymptomatic laboratory fish in this experiment and unfortunately do not know if they were infected by RMBV. We have modified our statements in the manuscript about the range of transmission and infection phenotypes that we have observed in laboratory and pet trade zebrafish naturally infected by RMBV. In summary, there are many intriguing possibilities that explain these data - variation in the dose of birnavirus particles at potential virus transmission events, differences in contact with infected pet store tank mates, and differences in immune status of the lab zebrafish are several possibilities we are considering and pursuing in future studies to advance the value of the co-housing model for virus discovery. We suspect that most viruses have a <100% transmission rate between individuals in a natural infection setting.

It is difficult to assess the virulence of birnavirus from our cohousing experiment alone. Not all fish developed lesions, but the fish that did develop lesions all contained birnavirus reads. We used a relatively-inefficient whole-transcriptome RNA sequencing approach to discover novel pathogens. Now that we know the sequence of the birnavirus, we can assay many more fish by developing a standardized qPCR assay instead of whole-transcriptome RNA sequencing. We now specify in the discussion that the range in disease phenotypes that we observe associated

with RMBV could result from differences in immunological life history, genetics, stage of infection, or a combination of influences. We hope to carry out controlled experiments with RMBV in the future to distinguish between these and other possibilities.

If pet shop fish are not hemorrhaging, how can authors know that pet shop fish are infected? Is infection strictly assessed by clear signs of hemorrhaging? What is causing hemorrhaging? Can non-hemorrhaging lab zebrafish (as in the case of pet shop) also have virus? Can the authors clarify whether they used any other criteria / measurement beyond haemorrhaging to assess zebrafish health eg. some scoring system which also looked at behaviour / swimming / more general health?

The data we collected from pet store and symptomatic lab fish cannot distinguish between two possibilities: 1) that only a fraction of lab fish were infected, i.e. transmission is <100%, as in most natural infections, or 2) that some lab fish are infected yet lack the most severe visual symptoms of infection.

As described above, we include data from a second cohousing experiment in a revised manuscript where we detected RMBV infection in an asymptomatic pet shop fish that did not transmit infection to cohoused laboratory fish. Furthermore, preliminary data from a third cohousing experiment (described above) suggest that laboratory fish can be infected by RMBV without exhibiting symptoms. These results highlight how little we understand about the potential causes of hemorrhaging associated with RMBV infection, which we acknowledge in the discussion. We also specify in the methods that no regimented health scoring system was used but that no qualitative signs of disease were observed other than hemorrhaging. One last piece of evidence that we add in the revised manuscript (Figure S1) is that although the RMBV-infected pet trade fish lacked visible signs of disease they nonetheless exhibited signs of antiviral responses at the gene expression level. In the modified discussion section we propose future experiments where general health and antiviral gene expression is monitored during RMBV exposures to better understand when infection leads to disease phenotypes or not.

How is virus transmitted? Is it fish-fish contact? Is there vertical transmission (at least in the pet shop colonies)? Can it be treated?

These are immediate questions when studying any new natural infectious agent. As this reviewer is no doubt aware, zebrafish offer a unique opportunity to address these questions. For example, we can co-house fish in tanks that do or don’t permit physical contact, cross pet store fish and measure viral load in the next generation, and/or attempt treatments with screens of FDA-approved drugs. We have added to the discussion to clarify that we observed horizontal transmission through an unknown route and agree that the possibility for vertical transmission is an important question.

As with the questions raised above, given the pathogenic outcomes of infection, we are waiting on approval of an updated IACUC protocol to design and execute followup experiments. Based

on our data across multiple pet stores, we speculate that RMBV is prevalent in pet stores and perhaps prevalent in the commercial hatcheries that supply most pet stores.

Reviewer #2: This manuscript describes a broad, sequencing-based approach to identifying new zebrafish pathogens taking advantage of the microbial (and pathogen) diversity of pet store obtained zebrafish. By sequencing, they are able to identify potential viral, bacterial and eukaryotic pathogens, using sequence similarity to guide them to potential pathogens of interest. Using this approach, the authors are able to characterize and reconstruct the genome of a novel birnavirus that the authors named Rocky Mountain birnavirus (RMBV). They demonstrate transmission of RMBV to naïve laboratory populations, resulting in hemorrhaging of infected populations and also investigate the inflammatory changes of infection. Overall, these experiments are well done and establish an interesting pipeline for the discovery of new pathogens of zebrafish that could potentially be applied to other fish species as well as other models. The identification of a new native pathogen of zebrafish would also be potentially useful for modeling of viral infection within zebrafish. These findings will be of interest to the zebrafish community and the fish immunology community and may also be of interest to immunologists in other model organisms. However, the central finding of the manuscript - the identification and experimental infection with RMBV requires some additional validation.

We thank the reviewer for their thoughtful comments and their interest in the present study.

Concerns:

For the experiments cohousing RMBV-infected pet store zebrafish with laboratory zebrafish, 3 zebrafish are found to have hemorrhage, it is unclear what the status of the other 7 cohoused zebrafish is. Are they also infected, but asymptomatic or are they uninfected? This should be tested for since the presence of asymptomatic carriage in their laboratory populations may change their interpretations, or if it is already known it should be described in the text.

We didn't have the sequence of RMBV in time to develop a rapid assay for viral load in cohoused fish. For the co-housing experiment, we had to rely on an unbiased RNAseq approach to discover pathogens in three tissues - intestine, whole kidney marrow, and spleen. Given the cost of such an experiment, and the uncertainty of what we might find if anything, we selected lab fish that displayed overt signs of pathology (hemorrhaging). All three of these fish were infected with RMBV. Unfortunately we do not know the infection status of the other seven laboratory fish that were asymptomatic, and have amended the text to make this clear – "RNA was not collected from the seven cohoused zebrafish that did not exhibit disease, and the infection status of these animals is unknown."

We now share the preliminary results from another ongoing co-housing experiment, in which pet trade fish from a different pet store source (also known to be a source of RMBV) were co-housed with laboratory fish. We did not see hemorrhaging in laboratory fish, but 1/4 fish had robust levels of RMBV suggesting transmission (see above, Reviewer Figure 1). In summary,

we believe that a spectrum of transmission, antiviral responses, and hemorrhagic symptoms are possible in a natural RMBV infection scenario.

In future studies of RMBV transmission we hope to develop improved quantification methods for examining infection status in many fish regardless of disease symptoms. We have updated the text and discussion to reflect the limitations in our understanding of the link between RMBV infection and disease.

RMBV is described as asymptomatic in pet store fish. However, one possible explanation is that symptomatic RMBV-infected fish had already died prior to purchase or due to disease, these animals weren't made available for purchase so instead only asymptomatic carriers were available. Is there any health data available from the pet store to support completely asymptomatic carriers? This possibility should be discussed, particularly if a percentage of laboratory zebrafish are also found to asymptomatically carry RMBV.

The possibility of pet trade fish succumbing to birnavirus infection in the hatchery or pet store is high. We have no information regarding mortality rates and causes of mortality from pet trade fish. Our attempts to discuss health records with pet store managers have been unsuccessful - it is not clear that records exist with this data and it is even less likely that pet trade suppliers would be willing to share this information with customers. The literature from birnavirus infections in salmonids, describes that birnavirus infections are most deadly in early life stages (see Dopazo CP 2020, reference 19 in the text). We suspect that the outcomes of encounters with RMBV depend on the life stage and previous infection history in zebrafish and mention this in the discussion. We also agree with the potential for asymptomatic RMBV infection in laboratory zebrafish and include this in the revised discussion and also described above in Reviewer Figure 1.

Reviewer #3: In the article "Microbe transmission from pet shop to lab-reared zebrafish reveals a pathogenic birnavirus", the authors describe a novel infectious pathogen that is transmissable between pet-trade zebrafish and lab-reared zebrafish. Their methods are clear and their conclusions are supported by their data. This is an appropriate level of mechanism for a Discovery Report. It leads me to ask many additional questions and get excited for the future of these sorts of analyses, as well as the data they have collected from this initial investigation.

We thank the reviewer for their enthusiasm about our study.

There are a few comments and questions that I would like to see addressed:

1) The authors use the terms metagenomic and metatranscriptomic fairly interchangeably, but it seems that the main work was done using materials derived from RNA extraction. Calling the experimental approach "metagenomics" made initial interpretation of Figure 1D perplexing.

We thank the reviewer for the clarifying suggestion and updated the text to metatranscriptomic throughout.

2) The sentences leading up to the sentence "These results underscore the use of zebrafish rom the pet trade as potential sources of viruses and parasites that are not commonly found in laboratory zebrafish" led me to a different conclusion. If we cannot distinguish between the lab/pet trade sources and there are similar bacterial communities in zebrafish found in labs and pet stores, then how is the pet trade a potential source of novel bacterial infections? I think there might just be a poorly worded sentence or the data from S4 leads us to believe that lab and pet stores have indistinguishable infectious bacterial agents. If the emphasis is on bacterial infections being similar compared to viral infections being unique, then that needs to be more explicitly stated.

This was a poorly worded section. What we intended to highlight was that bacterial communities were quite similar between lab and pet trade fish. Our principal component analysis did not indicate large differences between different groups of zebrafish - intragroup diversity was as great as intergroup diversity (now S5). By contrast, the viruses and eukaryotic parasites were very different. We have updated the text to clarify this point.

3) In the co-housing experiments, were the sexes of the fish taken into consideration? Was there any instance of fighting among the co-housed?

Laboratory fish of both sexes were placed in the cohousing tank. However, the three fish displaying hemorrhaging all happened to be male and these were the ones selected for RNA seq analysis. No fighting was observed among the cohoused fish and the tanks provided ample room for escape. We used 10 gallon tanks at a stocking density of 15 total zebrafish; by contrast laboratory fish are normally housed at a density of 25 fish in 0.5 gallon tanks). These details are now included in the methods section..

4) This may be beyond the scope of the work in this article, but are there different transcriptional responses in infected pet-trade zebrafish compared to the infected laboratory zebrafish? This would be additional support for the claims in the conclusion that cite the importance of "specific pairing of host and virus genomes" in the onset of disease. Given that the pet trade zebrafish were asymptomatic, do the authors believe that they had reduced inflammatory responses OR that they were survivors of a past infection and like IPNV, they are still able to be carriers?

We re-analyzed our data to investigate transcriptional responses to asymptomatic infections in the pet trade fish. Indeed, pet trade fish with birnavirus infections - those from pet shop 3 - have elevated expression of interferon stimulated genes, suggesting that their immune system is responding to an ongoing infection (Figure S1). An important caveat raised by the reviewer is that we don’t know where along the course of infection these fish stand, and we don’t have the power to detect differences in immune response between pet trade and laboratory-raised zebrafish. However, these data suggest a strong correlation between RMBV infection and antiviral defense responses, which can be disconnected from the visible symptoms we observe in the laboratory fish facing an acute infection. Future experiments could disentangle the hypotheses raised by the reviewer, pending approval of a more robust IACUC protocol.

5) Could the authors provide precise numbers for the symptomatic laboratory zebrafish in the co-housing experiments? What proportion developed lesions? What proportion had identifiable viral RNA in them?
Overall, this is a well-written and clear article. The authors do not oversell their claims and provide data-based support for nearly all of their conclusions. I look forward to seeing more zebrafish studies on host-RMBV interactions.

Three cohoused laboratory fish developed lesions. These three animals were dissected and RNA extracted and sequenced from intestine, whole kidney marrow, and spleen tissues. All three of these fish had birnavirus reads. The other seven co-housed lab fish did not exhibit overt signs of disease and were not sampled for RNA sequencing. At the time, we didn't have the sequence of RMBV to deploy a rapid assay for viral load in cohoused fish.

We now provide the results from a second, smaller scale co-housing experiment (Figure S2). In this experiment, two pet trade and two laboratory fish were co-housed for a month, and intestines sampled from all four fish for RNAseq. In this case, only one of the pet trade fish was positive for RMBV. Neither laboratory fish was positive for RMBV at the time of sampling. We now discuss these data in the main text, highlighting that "These results demonstrate that in some contexts, zebrafish can carry RMBV without transmitting infection to potential recipients in a shared environment."
